# Sensors vs. experts - A performance comparison of sensor-based fall risk assessment vs. conventional assessment in a sample of geriatric patients

**DOI:** 10.1186/1472-6947-11-48

**Published:** 2011-06-28

**Authors:** Michael Marschollek, Anja Rehwald, Klaus-Hendrik Wolf, Matthias Gietzelt, Gerhard Nemitz, Hubertus Meyer zu Schwabedissen, Mareike Schulze

**Affiliations:** 1 Hanover Medical School, Peter L. Reichertz Institute for Medical Informatics, Carl-Neuberg-Str. 1, Hanover, 30625, Germany; 2 University of Braunschweig - Institute of Technology, Peter L. Reichertz Institute for Medical Informatics, Mühlenpfordtstr. 23, Braunschweig, 38106, Germany; 3 Braunschweig Medical Center, Department for Geriatric Medicine, Gliesmaroder Straße 29, Braunschweig, 38106, Germany

## Abstract

**Background:**

Fall events contribute significantly to mortality, morbidity and costs in our ageing population. In order to identify persons at risk and to target preventive measures, many scores and assessment tools have been developed. These often require expertise and are costly to implement. Recent research investigates the use of wearable inertial sensors to provide objective data on motion features which can be used to assess individual fall risk automatically. So far it is unknown how well this new method performs in comparison with conventional fall risk assessment tools. The aim of our research is to compare the predictive performance of our new sensor-based method with conventional and established methods, based on prospective data.

**Methods:**

In a first study phase, 119 inpatients of a geriatric clinic took part in motion measurements using a wireless triaxial accelerometer during a Timed Up&Go (TUG) test and a 20 m walk. Furthermore, the St. Thomas Risk Assessment Tool in Falling Elderly Inpatients (STRATIFY) was performed, and the multidisciplinary geriatric care team estimated the patients' fall risk. In a second follow-up phase of the study, 46 of the participants were interviewed after one year, including a fall and activity assessment. The predictive performances of the TUG, the STRATIFY and team scores are compared. Furthermore, two automatically induced logistic regression models based on conventional clinical and assessment data (CONV) as well as sensor data (SENSOR) are matched.

**Results:**

Among the risk assessment scores, the geriatric team score (sensitivity 56%, specificity 80%) outperforms STRATIFY and TUG. The induced logistic regression models CONV and SENSOR achieve similar performance values (sensitivity 68%/58%, specificity 74%/78%, AUC 0.74/0.72, +LR 2.64/2.61). Both models are able to identify more persons at risk than the simple scores.

**Conclusions:**

Sensor-based objective measurements of motion parameters in geriatric patients can be used to assess individual fall risk, and our prediction model's performance matches that of a model based on conventional clinical and assessment data. Sensor-based measurements using a small wearable device may contribute significant information to conventional methods and are feasible in an unsupervised setting. More prospective research is needed to assess the cost-benefit relation of our approach.

## Background

It is well-known that fall events constitute an important factor with regard to mortality, morbidity and costs in our aging population. These events have a high incidence especially in the elderly: 25.1% of the men and 37% of the women aged 65 years and above fall at least once within 12 months [1]. The highest incidence is reported for geriatric inpatients [1], which often have several risk factors [2] at the same time and suffer from multiple diseases: The prevalence rate of five or more somatic diseases for persons aged 70 years and above has been reported in the Berlin Aging Study to be 88% [3]. As fall events and their consequences are very costly - an estimated annual 19.2$ billion in the U.S. [4] - preventive measures have been investigated intensively [5]. These measures themselves are costly, so that two predominant questions are: Who should be treated in the first place, and who should receive which kind of preventive measure?

In order to identify persons at risk to fall down - thus being eligible for preventive treatment - many risk assessment tools, e.g. the *Timed Up&Go *test (TUG) [6] or the *St. Thomas Risk Assessment Tool in Falling Elderly Inpatients *(STRATIFY) [7] have been developed and evaluated in a multitude of studies. Comprehensive reviews can be found e.g. in [2,8,9]. Several tests have also been used to predict falls in outpatients, often with a specific group of patients. Kikuchi et al. report that, in a prospective study with 79 patients having a diagnosis of cognitive impairment and lasting 12 months, only their fall-predicting score, a self-answered 21-item questionnaire, was predictive of future falls [10], but not e.g. the TUG. The latter test was, in contrast, found as the only predictive parameter for falls in patients after hip surgery in a 6-month prospective study by Kristensen et al. [11] Hale et al. found that mobility scores were not associated with falls in a 12-month prospective study with 120 geriatric outpatients, but history of falls was [12]. Oliver et al. conclude that even the best tools are not able to identify the majority of fallers [9]. Keeping this in mind along with the often time-consuming nature of fall risk assessment tests (e.g. the Performance-Oriented Mobility Assessment, POMA [13]) that frequently require expert knowledge, several research groups have developed the idea to perform a sensor-based automatic or semi-automatic assessment using wearable inertial sensors [14-17]. Apart from offering continuous and objective data, this approach may also serve to detect fall events once they have happened, being aware of the fact that many falls go by undetected and a person may lie injured hours or even days in her or his flat. Despite promising first results of this sensor-based approach developed by the authors [18], it remains unclear how well the new methods perform in comparison with conventional fall risk assessment tools.

Therefore, the aim of our research work for this paper is to examine the predictive performance of our new sensor-based method for fall risk assessment in comparison with conventional and established methods. The comparison is based on one-year follow-up data obtained in a prospective study.

## Methods

### General approach

We recruited a sample of geriatric patients - who are known to have the highest risk of falling [1] - and performed selected conventional assessment tests which are used to determine fall risk. Furthermore, fall risk was assessed by the interdisciplinary geriatric care team (physicians, nurses, physiotherapists, occupational therapists) of the *Department for Geriatric Medicine *at *Braunschweig Medical Center *in Germany. In addition to these measures, a sensor-based assessment of fall risk was performed, which employs gait and motion parameters obtained during a Timed Up&Go test and a 20 m walk during the patients' hospital stay. These parameters are: kinetic energy, pelvic sway along the transversal axis, standard deviation of gait periodicity, mean step duration, step length, number of steps during Timed Up & Go test and a number of spectral density distribution parameters such as the frequency of the most prominent spectral density peak. The sensor equipment used, the methods for parameter extraction from raw data and the generation of prediction models are explained in detail in [18,19]. The study protocol has been approved by the Hanover Medical School ethics committee.

### Study population

The study population consisted of geriatric inpatients of the Braunschweig Medical Center's Department for Geriatric Medicine who met the following inclusion criteria:

• admission between April 24^th ^and October 18^th^, 2007

• ability to stand up and walk

• written consent (also by a third party) to take part in the follow-up interviews

Although 119 patients took part in the first phase of the study, including the geriatric fall risk assessment tests and the sensor measurements, only 50 were able or willing to participate in the follow-up investigation (37 women and 13 men). The reasons for drop-outs were: death (n = 17), untraceable (n = 13), no answer to the letter asking for written consent (n = 27), rejecting the telephone interview despite previous consent (n = 4), and several other problems such as deafness or cognitive impairment (n = 8) [18]. Four of the motion sensor data sets were corrupted, so that altogether 46 persons (mean age 81.3 years) could be included in the fall risk assessment performance comparison study. With regard to the high number of drop-outs we compared the group of participants with the drop-outs in respect of relevant clinical parameters and fall risk assessments (age, body mass index, Timed Up&Go test, geriatric team risk score as described below, STRATIFY score and Barthel index score). There were no statistically significant differences between the two groups, except for a higher STRATIFY score in the drop-out-group (2.8 vs. 2.3, p = 0.036). As mental alteration is one of the STRATIFY items, this result may indicate that persons with cognitive impairments were more likely to decline to give written consent or even to die. In a further analysis of STRATIFY sub-items, however, no significant group differences could be identified.

### Conventional geriatric fall risk assessment tests and additional clinical parameters

Several clinical parameters as well as geriatric assessment tests were performed by clinical staff members on admission to the geriatric department. The clinical parameters were age, sex and body mass index (BMI). Furthermore, the *St. Thomas Risk Assessment Tool in Falling Elderly Inpatients *(STRATIFY) score [7] and the Barthel index [20] were assessed, and the *Timed Up&Go *test [6] was performed plus an additional 20 m walkway.

Apart from these standardized methods, the multidisciplinary geriatric care team assessed individual fall risk using a self-developed rating scale, which simply consists of three values: *no risk, low risk *and *high risk*.

In addition to the above-mentioned assessments which are performed during hospital stays, in the second phase of the study - conducted one year after the first phase - we used telephone interviews in order to assess fall events within the last year as well as general physical activity levels. A detailed account of physical activity levels among fallers and non-fallers can be found in [21]. The fall-related interviews were structured in accordance with the *Prevention of Falls Network Europe *(ProFaNE) consensus criteria [22] and are described in more detail in [18].

### Sensor equipment

**Figure 1:**
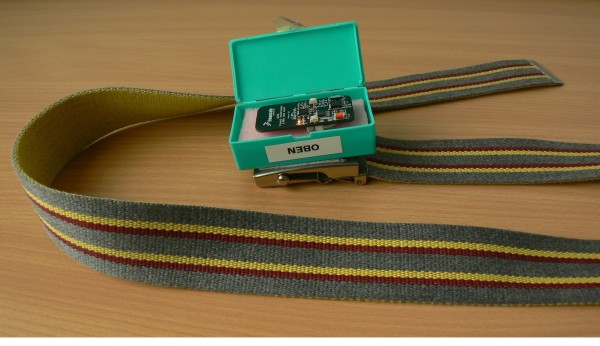
**Triaxial accelerometer sensor (*Freescale RD3152MMA7260Q*), sensor casing and belt**. No skin contact is necessary for the sensor function

During the inpatient phase of the study, all participants wore a wireless triaxial accelerometer system (*Freescale RD3152MMA7260Q*) on a belt around the waist (Figure 1) during the Timed Up&Go test and the 20 m walkway in the physiotherapy department of the clinic. The data were transmitted to a PC and stored for processing.

### Classification model induction and evaluation

In order to evaluate the predictive performance of the several fall risk assessment tools, we chose a two-fold approach. First of all, we used the two dedicated fall risk assessment scales - the STRATIFY score and the Timed Up&Go test - along with the care team score to distinguish between fallers and non-fallers within the year of follow-up. The cut-off points were set as proposed by the developers in their original publications: ≥ 2 points for the STRATIFY score [7], > 20s for the Timed Up&Go test [6] and *no risk *vs. *low/high risk *for the team score. For each score, we constructed a contingency table and calculated sensitivity (SENS), specificity (SPEC), positive (PPV) and negative predictive values (NPV) as well as overall classification accuracy (ACC).

In a second step, we used all clinical and fall risk assessment parameters to automatically induce a classification model (model CONV). The same has been done previously for the parameters that were extracted from sensor data and overall activity (model SENSOR) [18]. We chose to use a logistic regression model based on its known properties of stability and performance in small data sets and employed the Open Source toolkit *Waikato Environment for Knowledge Analysis *(WEKA, version 3.7.1, *simple logistic *algorithm; parameters: error on probabilities = true, heuristic stop = 50, maximum number of iterations for LogitBoost = 5000, use cross validation = true, no weight trimming) [23]. For model induction, the binary attribute *fall within the last year *(yes/no) was used. The multi-parameter data sets were pre-processed using a feature selection algorithm in order to exclude parameters with low information (WEKA, version 3.7.1, *wrapper subset evaluator*, employing the *simple logistic regression *algorithm as described above). All automatically induced classification models were evaluated using a ten times ten-fold cross-validation procedure, and SENS, SPEC, PPV, NPV, ACC and the area under the curve (AUC) were calculated. Furthermore, we computed the each model's Brier score as a performance measure [24], which is the mean squared difference between the observed outcome and the predicted probability for each instance in the data set. Finally, the positive likelihood ratios (+LR) of all models were calculated as an additional performance measure that spans the whole of each contingency table.

## Results

Tables 1, 2 and 3 show the results of the single fall risk assessment tests for the prediction of actual fall events within a year after discharge from the geriatric ward. In Table 4, all +LR values are presented. The STRATIFY score (Table 1), a dedicated fall risk tool, has an overall classification accuracy of 48% with a good sensitivity of 79% but a low specificity of 26%. While the NPV is 63%, the PPV is only 43%, meaning that a positive assessment result does not predict actual falls well.

**Table 1:** Classification results and contingency table for the STRATIFY score [7] (cut-off point ≥ 2 points)

STRATIFY score					
			**contingency table**		

classification accuracy	48%		fall within one year		

sensitivity	79%		yes	no	Sum

specificity	26%	pred. yes	15	20	35

negative predictive value	63%	pred. no	4	7	11

positive predictive value	43%	sum	19	27	46

**Table 2:** Classification results and contingency table for the Timed Up&Go test [6] (cut-off point > 20s)

Timed Up&Go test					
			**contingency table**		

classification accuracy	50%		fall within one year		

sensitivity	90%		yes	no	Sum

specificity	22%	pred. yes	17	21	38

negative predictive value	75%	pred. no	2	6	8

positive predictive value	45%	sum	19	27	46

**Table 3:** Classification results and contingency table for multidisciplinary geriatric team fall risk score (4 missing values)

TEAM assessment					
			**contingency table**		

classification accuracy	55%		fall within one year		

sensitivity	63%		yes	no	Sum

specificity	50%	pred. yes	10	13	23

negative predictive value	68%	pred. no	6	13	19

positive predictive value	44%	sum	16	26	42

**Table 4:** +LR values of all five classification models including the confidence intervals

model name	+LR value	95% confidence interval
STRATIFY score	1.07	0.71-1.61

Timed Up&Go test	1.15	0.83-1.59

Team Assessment	1.25	0.63-2.49

model CONV	2.64	1.07-6.5

model SENSOR	2.61	0.94-7.26

The *Timed Up&Go *test results in Table 2 show an overall classification accuracy of 50%, where a high sensitivity of 90% is pitted against a very low specificity of 22%. Similar to the STRATIFY score results, the NPV is slightly higher (75%) than the PPV (45%).

The geriatric team's fall risk assessment score (Table 3) shows more balanced, though not really much better results: A classification accuracy of 55% is accompanied by a sensitivity of 63% and a specificity of 50%. The NPV is 68% and the PPV is 44%. The +LR values (Table 4) of all three simple fall risk assessments (1.07, 1.15 and 1.25) confirm their low predictive power, yet among these the team score has the highest hit ratio.

The automatically generated classification model CONV (Table 5) based on clinical and geriatric assessment data shows markedly better performance values than the three previous tests: The classification accuracy is 72% with a sensitivity of 68% and a specificity of 74%. Furthermore, both the NPV (77%) and the PPV (65%) are balanced and on a fair level. The overall good performance of this model is also shown by both the Brier score of 0.2, an AUC of 0.74 and a statistically significant +LR value of 2.64 (Table 4).

**Table 5:** Classification results and contingency table for logistic regression model based on clinical data and fall risk assessment tests

model CONV					
classification accuracy	72%				

sensitivity	68%		**contingency table**		

specificity	74%		fall within one year		

negative predictive value	77%		yes	no	Sum

positive predictive value	65%	pred. yes	13	7	20

Brier score	0.20	pred. no	6	20	26

AUC	0.74	sum	19	27	46

The classification model SENSOR (Table 6, [18]) matches the CONV model in its measures: Classification accuracy is 70%, with a sensitivity of 58% and a specificity of 78%. NPV (72%) and PPV (65%) are also level. The +LR value of 2.61, however, does not reach statistical significance due to the broader confidence interval.

**Table 6:** Classification results and contingency table for logistic regression model based on sensor data and long-term physical activity level

model SENSOR					
classification accuracy	70%				

sensitivity	58%		**contingency table**		

specificity	78%		fall within one year		

negative predictive value	72%		yes	no	Sum

positive predictive value	65%	pred. yes	11	6	17

Brier score	0.21	pred. no	8	21	29

AUC	0.72	sum	19	27	46

## Discussion

The performances of the simple fall risk assessment tools used in this study - the STRATIFY score, the Timed Up&Go (TUG) test and the geriatric care team rating - are limited. In a recent meta-analysis Oliver et al., who have developed the STRATIFY score, report the following values for geriatric patients: SENS 67.2%, SPEC 51.2%, PPV 23.1% and NPV 86.5% (n = 1285 patients, four different studies) [9]. Kim et al. have also evaluated the STRATIFY score, albeit with a much younger cohort (mean age 56 years, n = 5489 patients, 60 fallers), and find: SENS 55%, SPEC 75.3%, PPV 2.4% and NPV 99.3% [25]. Our results show a slightly worse performance than was reported in the meta-analysis by Oliver et al. [9]. This may well be due to our very small sample size. The same applies to the Timed Up&Go test. Nordin et al. have studied the predictive validity of the TUG in 183 patients with a mean age of 84.3 years and a cut-off point of 20s [26]. They report a sensitivity of 79% and a specificity of 32%. In the large *Tromsø study *Thrane et al. find sensitivity values of 44-14% and specificity values of 58-90% for the TUG, depending on the choice of the cut-off points (here between 12 and 17s) [27]. Kristensen et al. report SENS 95%, SPEC 35%, PPV 41%, NPV 93%, +LR 1.5, -LR 0.1 for the TUG's predictive performance for patients after hip surgery (mean age 81 years, n = 59 patients, 19 fallers, cut-off value 24s) [11]. Our results (Table 2) also show a remarkable sensitivity of 90% for the TUG, yet the specificity is way too low for a screening test. This is confirmed by the low +LR value of 1.15.

Both tests are very simple to perform, either by history taking or by conducting a simple physical test, and both take only a couple of minutes. Therefore, these tests may serve well - and in fact are frequently used - as general screening methods, if necessary followed by more complex, multimodal assessment inventories such as the *Physiological Profile Assessment *(PPA) [28].

The geriatric care team fall risk score may be perceived as a very subjective measure, yet it represents the professional opinion of several experienced experts that is very likely based on an intuitive understanding of the complex concept 'fall risk' as well as on a multitude of observations of a certain patient. This solid foundation is reflected by the fair performance values of this score, which are the most balanced of the three simple tests. Similar results have been found in [26], where 'global rating of fall risk' (*low/high*) by staff members achieved a sensitivity of 56% and a specificity of 80%.

The automatically induced model CONV (Table 5) shows better performance values than all of the above tests. This is of course due to the approach of including basic clinical data such as sex, BMI and age in the induction process, but also to the combination of different assessment methods ranging from a physical test (TUG) over a measure of daily activity capability (Barthel index) to a fall risk score (STRATIFY). In the induction process, the most relevant parameters or scores are identified and included, so that performance is optimized. The multitude of candidate parameters may capture the multi-factorial concept of fall risk more adequately than a single test. The performance measures show that CONV can identify most of the fallers and non-fallers correctly, based on their one-year outcome. Thus, this model could be suitable as a screening test for geriatric patients, facilitating the prescription of preventive measures.

When compared to the previously computed SENSOR model, which is based merely on accelerometer sensor data and overall activity levels, we can state that this model performs almost equally well than the CONV model. The Brier scores (0.21 vs. 0.2) are nearly the same, as are the AUC (0.74 vs. 0.72) and +LR values (2.64 vs. 2.61). Therefore, regarding our preliminary results we may conclude that by using sensor data - which may be recorded over extended periods of time during normal daily activities with a small and unobtrusive device - we can match the performance of conventional methods with regard to fall risk assessment in a sample of geriatric patients. The advantage of our approach is of course the absent necessity of an expert physiotherapist, nurse or physician to perform the assessment. This could be done by the wearable device itself, using long-term motion data along with the developed algorithm. Potential drawbacks of our approach are possible technical failures such as data loss from the accelerometer device, acceptance issues, limited battery lifetime and the current lack of technical infrastructures (e.g. sensor-enhanced health information systems [29,30]). Technical equipment and infrastructures are of course costly, but so are falls and their consequences in the first place.

Future prospective studies will have to be conducted with more patients and over an even longer period of time, evaluating the validity of our approach and our preliminary results in an independent patient sample on the one hand and the cost-benefit relation on the other hand. From an ethics point of view, however, one might argue that every fall that is avoided is a big benefit for the individual.

From a technical point of view, more research work is needed to look into the potential predictive parameters that can be extracted from sensor data [31]. Furthermore, in this study we have not considered any information from the patients' electronic health records (EHRs) [32], such as diagnoses or additional history. Considering the multi-factorial aetiology of falls [33], our sensor-based information may well be used in combination with resp. as supplement to conventional geriatric assessment tools and other clinical data.

## Limitations

Our sample size is small and this - in comparison with large trials evaluating the conventional methods-limits the generalizability of our results. So does the fact that, due to the sample size, we cannot use separate training and test data sets for model induction. Nevertheless, we have chosen a well-established procedure to avoid over-fitting of our models, namely ten times, ten-fold cross-validation. The necessity for written consent to be returned by the patients via surface mail may have led to the exclusion of persons with cognitive impairments, even though consent by a third party was an option. Furthermore, in our follow-up study telephone interviews were used to identify fall events. This approach is error-prone, as many factors (e.g. cognitive impairment) may affect the recall of such events. In addition to this, considering the group of patients, within a period of twelve months risk factors may have changed significantly. Therefore, daily recordings as well as more frequent interviews, e.g. on a monthly basis as recommended in ProFaNE consensus criteria recommendation no.7 [22], might have reduced the error rate, but have not been performed due to a lack of resources. Hence, in our future prospective studies, we will include a more stringent monitoring approach.

From an economic perspective, it remains unclear if the prediction results are good enough to justify the implementation of costly preventive measures for the false positives [5]. A cost-benefit analysis should be conducted, comparing direct and indirect costs of fall events with those of preventive measures. Furthermore, despite promising preliminary studies (e.g. [34-36]), the patients' acceptance of long-term monitoring should be assessed, e.g. using the *Sensor Acceptance Model *[37].

## Conclusions

To the authors' knowledge, this is the first study to compare sensor-based fall risk assessment to conventional assessment tools in a prospective long-term setting. Our preliminary results indicate that a fall risk model based on accelerometer sensor data performs almost as well as a model that is derived from conventional geriatric assessment data. Therefore we may conclude that such a model can provide relevant information and thus - considering the multi-factorial aetiology of fall events - could be valuable not as a replacement of professional assessment scores and tools, but as a supplementary method which is feasible to be used outside of a supervised clinical environment.

## Competing interests

The authors declare that they have no competing interests.

## Authors' contributions

MM supervised the study, carried out the data analysis and drafted the manuscript. AR designed and conducted the telephone interviews. KHW participated in drafting the study protocol. MG conducted the sensor data measurements and participated in the data analysis. GN performed the geriatric assessment tests and participated in drafting the study protocol. HMZS conceived of the study and participated in its coordination. MS participated in the data analysis and the computation of performance values. All authors read and approved the final manuscript.

## Pre-publication history

The pre-publication history for this paper can be accessed here:

http://www.biomedcentral.com/1472-6947/11/48/prepub
